# Empathy in Human–Robot Interaction: Designing for Social Robots

**DOI:** 10.3390/ijerph19031889

**Published:** 2022-02-08

**Authors:** Sung Park, Mincheol Whang

**Affiliations:** 1 School of Design, Savannah College of Art and Design, Savannah, GA 31401, USA; spica7601@gmail.com; 2 Department of Human Centered Artificial Intelligence, Sangmyung University, Seoul 03016, Korea

**Keywords:** human–robot interaction, social robot, virtual human, empathy, affect, emotion

## Abstract

For a service robot to serve travelers at an airport or for a social robot to live with a human partner at home, it is vital for robots to possess the ability to empathize with human partners and express congruent emotions accordingly. We conducted a systematic review of the literature regarding empathy in interpersonal, virtual agents, and social robots research with inclusion criteria to analyze empirical studies in a peer-reviewed journal, conference proceeding, or a thesis. Based on the review, we define empathy for human–robot interaction (HRI) as the robot’s (*observer*) capability and process to recognize the human’s (*target*) emotional state, thoughts, and situation, and produce affective or cognitive responses to elicit a positive perception of humans. We reviewed all prominent empathy theories and established a conceptual framework that illuminates critical components to consider when designing an empathic robot, including the empathy process, outcome, and the *observer* and *target* characteristics. This model is complemented by empirical research involving empathic virtual agents and social robots. We suggest critical factors such as domain dependency, multi-modality, and empathy modulation to consider when designing, engineering, and researching empathic social robots.

## 1. Introduction

Interest in empathic robots is growing in academia and industry. Softbank’s Pepper is designed to understand a human’s mood [1] and respond accordingly, which requires both emotion recognition and an expression engine. The long-awaited social robot Jibo was released in the market in 2018 with a range of social skills, including identifying family members and calling by names, telling jokes, and dancing. While interacting with such robots may certainly be entertaining, it is still early to say that state-of-the-art commercialized robots can *empathize* with humans.

We feel similar emotions as others, which is sometimes a result of understanding others’ thoughts and feelings. Empathy involves “an affective response more appropriate to someone else’s situation than to one’s own” [2]. Empathy considers the other’s affective state and situation, which leads to cooperation, prosocial behavior, altruism, and a positive relationship [2,3,4,5]. It seems critical for robots to *empathize* with human partners, that is, recognize human emotional states, thoughts, and situations, and behave accordingly in order to live with human partners at home, to help with their mental or health-related problems, or to assist their daily activities.

Researchers of human–robot interaction (HRI) have recently started exploring different aspects of empathy (for a survey, read [6]). The current state of research is far from achieving full-fledged empathetic capability, but recent progress in social and developmental psychology, neuroscience, and virtual agent research have highlighted research directions for empathic social robots.

The purpose of this review is twofold: (1) to understand the effects of robotic empathy on humans and (2) to identify the components necessary to design and engineer empathy for robots. Apparently, these two understandings inform each other. Researchers may also identify gaps in research and gain insights into establishing a research agenda.

To this end, we systematically selected literature on empathy in interpersonal, human–agent, and human–robot interaction. We then provide a working conceptual model of empathy applicable to HRI, based on the literature on social, developmental, and clinical psychology and neuroscience. This model comprises empathy processes, outcomes, and modulator factors of empathy. We then review the literature on virtual humans and social robots to extend our model of empathy. While the conceptual model of empathy is certainly not a computational model for empathic robots, it may provide a blueprint for a cognitive–affective architecture for engineers.

A general design guideline for empathic robots will be provided to inform designers about the elements required to engineer empathic robotics. Our review identifies that, depending on the purpose, context, and tasks of a social robot, critical factors of empathy to implement may vary. We outlined three types of empathic robots as a function of the complexity of the empathic process.

## 2. Methods

We referred to the most recent meta-analysis on social robots by Duradoni et al. [7] when establishing a search strategy for a systematic analysis. We limited the search terms to include “empathy” in conjunction with critical keywords related to interpersonal interaction (“dyadic”, “social”, “interpersonal”), and human–agent interaction (“embodied conversational agent”, “virtual humans”, “avatars”, “agents”), and human–robot interaction (“social robots”, “HRI”, “robots”).

We defined our inclusion criteria to be literature that is: (1) a paper published in a peer-reviewed journal or a conference proceeding, (2) written in English, (3) published until 2021, (4) an empirical study.

We used databases of Google Scholar, PsychArticles, PsychInfo, PubMed, Science Direct, and Sociological Abstracts. Table 1 includes the number of articles considered in our systematic review. The initial screening of the abstract resulted in 1116 (interpersonal interaction), 128 (human–agent interaction), and 76 (human–robot interaction). We excluded 188 articles based on the following exclusion criteria: (1) *Interpersonal literature*: the definition of empathy is identical to the original study, the paper does not add substantial findings to the literature, the article applies only to a limited domain; (2) *Human–agent and Human–Robot literature:* the manuscript adopted a loose folk-definition of empathy, the research investigates whether the participants empathize with the system and not an empathic system, the paper has critical flaws (e.g., low statistical power). As a result, we selected 70 (interpersonal interaction), 10 (human–agent interaction), and 12 (human–robot interaction) articles for a review.

## 3. Empathy in Interpersonal Interaction

The origin of *empathy* can be traced to the German term *Einfühlung*, which connotes the observer’s projection to the physical object of beauty. Lipps [8] later adapted this concept to understand other people. The English term *empathy* was coined by Titchner [9] as a translation of *Einfühlung*.

Empathy research has been conducted in the fields of social [10], developmental [11], and clinical psychology [12], and later neuroscience [13]. Since the discovery of mirror neurons in monkeys [14], neuroscientists have identified underlying neurological evidence for empathy [15]. Overlapping brain patterns were observed when an observer perceived the same emotions from a target, suggesting shared affective neural networks [16,17,18].

However, there is no consensus on the definition of empathy. The number of definitions is proportional to the number of researchers [19]. Scholars agree that empathy consists of multiple subcomponents [2,20,21]. A few critical elements of empathy are commonly identified across definitions (for an extensive review of empathy as a concept, see [22]). This review organizes prominent views on empathy in interpersonal research and suggests a comprehensive definition of HRI. Our definition has two functional roles: (1) deciding which empathy literature to include or exclude for our review and (2) establishing the cornerstones of the conceptual model of empathy.

The cognitive and affective aspects of empathy are probably the two most discussed topics in this field of study (see Table 2). Empathy definitions are organized into three groups: definitions with emphasis on (1) affective, (2) cognitive, and (3) both aspects of empathy. Only the original definitions are considered; that is, definitions that are mechanically combined are excluded. From this point on, all critical elements of empathy that merit their inclusion in the conceptual model are in *italics.*

Many researchers argue that empathy has two components: affective and cognitive [23]. Affective empathy generally connotes the *observer*’s visceral reaction to the *target*’s affective state. Cognitive empathy involves taking the *target*’s perspective and drawing inferences about their thoughts, feelings, and characteristics [24]. Several researchers exclude or conditionally include cognitive aspects in the definition of empathy. For example, Zaki [25] claimed that *perspective taking*, a cognitive process, is only regarded as a part of empathy when it involves experience sharing. Proponents of a narrower view also argue the difficulty in pinpointing the nature of the automaticity of empathy with the inclusion of cognitive components [15].

In our research, we adopted an inclusive approach and acknowledged both aspects of empathy. This is to avoid confining HRI research to only *motor mimicry* or emotion contagion and to include extensive empathic interaction based on cognitive elements such as context, past experience, and knowledge about the dyadic. In other words, to establish rapport and relationship with a human partner, a social robot requires both affective and cognitive aspects of empathy. As such, recent HRI research incrementally includes research on *perspective taking,* a form of cognitive empathy, of social robots [36].

As shown in Table 2, except Davis [20], most definitions stress the outcome of empathy. While most define empathic responses as similar or congruent [26,27,37], a few narrower definitions denote feelings identical to those of the *target* [28]. The empathic outcome is certainly a result of the empathizer’s or *observer*’s internal empathy process. A clear division of process and outcome is critical for specifying the causal relationship between the two. This specificity is required to engineer empathy through the cognitive architecture of a social robot.

One important aspect of empathy in HRI is its purpose. Social robots are designed with a particular purpose in mind, so the architecture of the empathic process should be designed to serve its goal. Given this, we define empathy for HRI as the robot’s (*observer*) capability and process to recognize the human’s (*target*) emotional state, thoughts, and situation and to produce affective or cognitive responses with the purpose of eliciting a positive perception of humans. The human perception of an empathic social robot ranges from liking, trust, and intention for long-term use, which we will address later.

**Figure 1:**
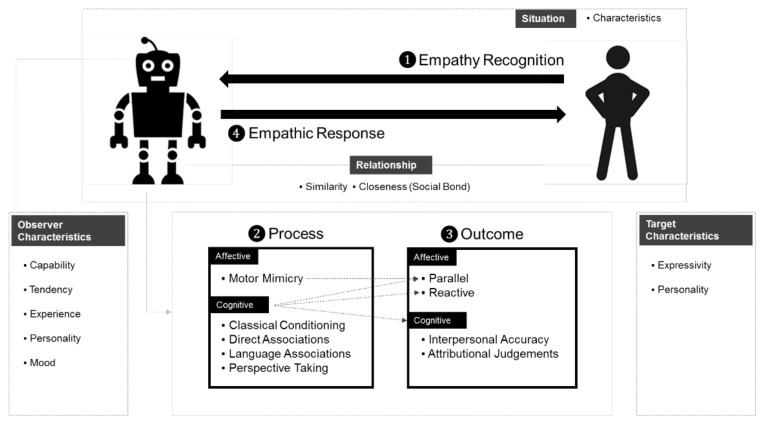
Conceptual model of empathy of HRI.

A review of the current literature resulted in a working conceptual model of empathy (see Figure 1). This model outlines the processes and critical components of empathy that are applicable to the design of social robots. It is an intermediate model to be evolved, with a review of empathy research on virtual agents and HRI. Construct elements and interaction processes are identified as comprehensive to include representative empathy scenarios involving social robots.

The assumption is that for humans to perceive a robot’s empathy positively (e.g., increased liking and trust), the robot’s empathy should be engineered similar to humans’ processing of empathy. As robots and humans are essentially different, elements of human empathy that have little value in HRI were excluded. Therefore, we emphasize that this is not an integrated model for understanding human interpersonal empathy, but rather a selective and organizing model to design an empathic social robot.

A typical empathic episode is initiated when *the observer* (robot) perceives empathic cues (expression or situation) from *the target* (human) through verbal or nonverbal channels (❶ in Figure 1). The observer then engages in an internal affective or cognitive *process* (❷). This results in the observer’s internal *outcomes* (❸). The observer may decide to express the emotional state or behavior to the dyadic *target*. If done so, an empathic response is given to the target (❹). We deliberately separated ❸ and ❹ as first suggested by Davis [34] in his interpersonal empathy model. This includes scenarios in which a robot empathizes with a human but keeps the emotions to itself, deciding not to express them at the moment. *The characteristics* of and the *relationship* between the observer and target, and the *situation* as to where, when, and what kind of empathic event occurred are the modulating factors that influence the processes. A detailed account of each element follows, with an explanation of the process (❷) and outcome (❸) preceding the empathy recognition (❶) and response (❹).

### 3.1. Processes

Empathy processes are the underlying mechanisms that produce empathy outcomes. We integrated and identified the most prominent empathy theories [2,21,34,38,39] and organized them into affective and cognitive processes. Each process has differential neural straits [15]. The two mechanisms merit different routes for empathy in an emotion–cognitive computational module because they may lead to different empathic outcomes.

**Motor mimicry**. This refers to the *observer*’s automatic and unconscious imitation of the target. Mimicry was first described by Lipps and organized by Hoffman [40] into a two-step process: (1) the *observer* imitates the *target*’s empathic expressions (e.g., facial expression, voice, and posture); (2) this imitation results in afferent feedback that produces a *parallel effect* congruent with the *target*’s feedback, as depicted in Figure 1. For example, a robot may imitate a human’s facial expression, who looks cheerful, and changes its emotional state accordingly. This mechanism is also referred to as *primitive emotional contagion* [41] or *the chameleon effect* [42]. Mimicry is important in building rapport [43] and makes the *observer* more persuasive [44]; however, under certain situations, this may produce a diminishing effect.

**Classical conditioning**. occurs when a neutral stimulus (NS) is repeatedly paired with an unconditioned stimulus (US), leading to an unconditioned response (UR). Once conditioned, the sole conditioned stimulus (CS) is sufficient for the *observer* to exhibit a conditioned response (CR). Similarly, empathy can occur when the *observer* pairs the empathic cues (NS) of the target with his or her emotional cues (US) and the associated affective state (UR) [2,45]. Such cues are not limited to the target’s facial expressions, but also include the *situation* and context in which empathic interactions occur [46].

For example, a family may have several members, with each member following their different schedules. Nevertheless, when they gather together in the evening or on weekends, they have a joyful time, full of positive emotions. A social robot may learn this connection between family members gathering together (NS), the positive facial expressions of family members (US), and the corresponding positive emotions for a robot (UR). Once conditioned, a social robot may expect and prepare services congruent with family gatherings (taking photos and dancing). However, *conditioning* is probably the least studied empathy process in the HRI.

**Direct associations.** When the *observer* perceives the target’s empathic cues (❶ in Figure 1), the observer feels the emotions attached to it if they match the observer’s past experience [38]. This is the general version of classical conditioning [20]. Social robots may have episodic memories with associated emotions and use them to “feel” the current situation. For example, a robot may visually recognize two people hugging and then draw from its past experience involving a hug that includes warm, nurturing, and calm emotions.

**Language associations**. Sometimes, empathy is the result of a language-based cognitive network that triggers an observer’s emotional state [38]. Language-mediated association does not require direct observation and is considered a more advanced cognitive process [20]. Eisenberg et al. [47] explained a similar process, dubbed an elaborated cognitive network.

This process typically involves a conversation or dialogue with a social robot. A target human may tell the robot that he or she went to a party the previous night. The word “party” may trigger the robot’s language network and its past emotions in a party. Technologically, language-mediated associates require the social robot to have automatic speech recognition (ASR) and natural language processing (NLP), and a semantic map of words associated with emotions.

**Perspective taking**. *Perspective taking or role taking* is considered the most advanced cognitive process among empathy processes. This involves the observer’s effortful process of imagining the target’s perspective and suppressing the observer’s perspective [2]. This advanced cognitive process alongside language-mediated association is what many researchers call *cognitive empathy* [34].

A robot should project an imaginary situation and state of the observer to mimic perspective taking, which humans do with considerable effort. For example, one fine morning, a robot may greet a human target who has a tired look on the face and may ponder why this person looks tired. It may consider several reasons; for example, it may consider that this being exam week, the human target may have underslept, which may cause it to state: “I hope you are not jeopardizing your health. You may be stressed about the exam, but sleeping well is also necessary to study well.” This is an empathic concern (*reactive outcome*). All of this requires a virtual construction of the target’s situation and the emotional states associated with it from the robot’s side.

The difference between the first three (motor mimicry, classical conditioning, direct associations) and the latter two (language associations, perspective taking) categories is whether the observer directly perceives the empathic cues. The latter two can be invoked without directly perceiving the target’s empathic cues (❶ in Figure 1).

### 3.2. Outcome

The empathy process of an observer yields affective or cognitive *outcomes.* Affective outcomes are further divided into parallel and reactive outcomes [34]. Parallel outcome indicates the matching of the observer’s emotion to the target’s and has been the focus of early empathy research [45,48]. The matching of emotion denotes an affective outcome congruent with the target, as suggested by many researchers [26,38,49]. Motor mimicry or classical conditioning may lead to a parallel outcome.

Empathy outcomes sometimes go beyond similar or congruent reactions that do not reproduce the observer’s state. Reactive outcome involves the observer’s emotion, which is different from the target’s [50]. Feelings falling under this category include sympathy [51], empathic concern [52,53], empathic anger [54], and personal distress [47].

Outcomes may be primarily cognitive, such as interpersonal accuracy—that is, an estimation of the target’s thoughts, feelings, and characteristics [34]. Insights are also regarded as the product of the cognitive empathy process, typically by *perspective taking* [30]. This ability depends on the empathic *capability* of the observer. In counseling psychology, the result of perspective taking does not necessarily imply emotional ties with the client [55].

Another important aspect of interpersonal accuracy applicable to HRI is its anticipatory nature [32]. A social robot may project many imaginary scenarios constructed from the target’s empathic cues, with each scenario anticipating the target’s behavior. A social robot may weigh in each scenario considering factors such as *context* and *past history* of the observer and may suggest helpful services to the human observer (e.g., turning off lights and playing classical music).

Much more technological advancement for a social robot is required to make *attributional judgments*, which refer to identifying the causes behind the target’s thoughts, feelings, and characteristics [34]. Human empathizers generally attribute causes to the observer’s situation rather than attributions [56]. Human empathizers tend to identify dispositional attributions as a cause of the target’s success and situational attributions for the target’s failure [57].

### 3.3. Observer and Target Characteristics

Many factors modulate the empathy process. First, the characteristics of the observer, target, and relationship between the two play an important role.

Empathy requires an observer’s *empathic capability* and the ability to perform the empathic process. This requires all elements of Figure 1 to recognize empathic cues, process empathy, and express empathic responses to the target. This is a stable characteristic of human observers. We included such characteristics for HRI because of its evaluative value. Similar to the Turing test, an empathy test for a robot is required to calibrate a social robot for empathy capability. This test identifies the strengths and weaknesses of many aspects of empathy and reveals whether the robot is appropriate for a specific social task in a certain setting. Designers and engineers may view this as the goal to be attained. To the best of our knowledge, there is currently no measurement to evaluate a robot’s empathic capability.

However, just because the observer is capable does not necessarily mean that he or she is likely to empathize with an empathic event. *Empathic tendency* involves an individual’s predisposition to engage in empathic interactions. This is a stable characteristic for humans, and self-reported measures have been developed to evaluate it [20,58,59]. For a social robot, we view *empathic tendency* as a more fluid variable to be manipulated. In the movie *Interstellar*, the robot TARS’s humor level could be changed by its human counterpart. Given that the need for empathy depends on the situation, the willingness to empathize or not has its own merit. This is especially important for social robots that are expected to interact with individuals in different situations (i.e., domain-independent), which we see as the final step of an empathic social robot (i.e., Type III empathic robot).

The accuracy of empathic response can only be ensured when targets’ *expressivity* enables their thoughts and emotions to be perceived [60]. In other words, a social robot cannot empathize if the human user does not express emotional cues (❶ in Figure 1). For designers of HRI, this means that empathy scenarios have to be carefully designed so that human users are led to express such cues without giving the impression that one is forced to do so (e.g., directly asking how one feels).

*Past experiences* of the observer are relevant because many cognitive empathic processes relate to past memory [61]. The HRI research continues to maintain and operate a memory-like structure for empathy, even though their domains and scenarios are limited.

The mood and personality of observers (robots) are also important modulating factors [15]. Given that the result of empathy affects one’s emotion, a social robot may have an emotion module embodying the empathy process. There is a close relationship between emotions and personality. Emotions are temporary, and personality remains stable over a long period of time [62]. A robot’s personality has long been researched [63,64] yet, to our knowledge, no interaction with empathy exists.

Finally, there is a clear female superiority in empathic capability due to raising and nurturing children [65], and a recent HRI study has revealed a few interesting observations with differential *gender* effects in interaction with a social robot [66].

### 3.4. Relationship

Characteristics such as similarity and social bonds are a joint function of both the target and observer [2,15,34]. The stronger the observer–target *similarity*, the stronger the likelihood and the intensity of the observer’s *empathic response* (❹ in Figure 1). Specifically, Cialdini et al. [53] demonstrated that empathy stemming from similarity is related to a sense of self–other overlap, an emotional signal of oneness. Additionally, observers tend to empathize more with targets with similar personalities and values [67]. *Familiarity* is also suggested to modulate the strength of an empathic response [2,15].

Given their similarity and familiarity, humans tend to be more empathic toward individuals with whom they have social bonds, including *friends* and *family members*, than strangers [68]. The type of relationship affects the type of prosocial action taken by the observer [2]. Social robots may bear similar empathy to certain events but behave differently with people according to relationships.

### 3.5. Situation

All empathic interactions occur within a specific situational context or behavior. In the HRI, this indicates the type of robot’s tasks and goals involving the empathic processes as well as where and when such interactions occur. *The observer’s* contextual appraisal has been argued in interpersonal research and physiopsychologically analyzed [15], suggesting several modulatory factors.

Not all empathic events were equally treated. Davis [34] emphasizes the *strength of the situation,* which influences the power of empathic responses in interpersonal interactions. A helpless target suffering from a traumatic event tends to produce a powerful empathic outcome. A social robot may tag priorities on empathic events and modulate empathic responses accordingly.

Empathy is also moderated by the target’s behavioral *characteristics*. Based on neurological evidence from a functional magnetic resonance imaging study, Singer et al. [69] found that men empathize more with people with fair social behavior. Lamm et al. [70] revealed that observers had a smaller empathic response to pain-afflicted targets when cure was justified.

### 3.6. Empathic Recognition (❶)

If a social robot is viewed as an information processor, empathic recognition is the input stage, and the empathic response (❹) is the output stage. This stage involves the characteristics of empathic cues and the type of modality used to recognize such cues. Humans utilize a full range of interpersonal modalities, including verbal and nonverbal communication channels. The verbal channels primarily involve speech or dialogue, which directly—but not exclusively— connect to language-based cognitive networks in the empathic process (❷). Nonverbal channels involve gait, posture, gestures, movement, distance, facial expressions, gaze, and physical factors (e.g., touch), as well as nonsemantic voice features (e.g., speech speed, pitch, variation) [71]. Many lead exclusively to motor mimicry (❷).

Through such modalities, the *target* releases empathic cues. We empathize more with primary emotions (e.g., happiness and sadness) than with secondary emotions (e.g., jealousy) [15]. Strong negative cues may elicit stronger observer responses [34]. This is also introduced as *the intensity* and *saliency* of the observed emotion [2,15]. For example, the intensity of the target’s pain-induced facial expressions modulates the observer’s empathic response [72]. This is due to the increasing or decreasing attention to an empathy-eliciting stimulus [15]. The effect is further moderated by *target characteristics* (e.g., a helpless person).

### 3.7. Empathic Response (❹)

Empathic interaction involves a constant parallel interaction between recognition and response. Empathic responses are directed toward the target, and the factors are nearly identical to recognition, including the *modality* and extent to which empathy is expressed.

However, higher-level constructs should be addressed, such as the purpose of expressing empathic responses. One purpose may be to build rapport and improve social relationships with human partners. On the other hand, a social robot may not be interested in building a long-term relationship but aim at increasing liking and trust through helping and caring behavior. As a social robot operates for a specific purpose, task goals should be outlined to select the modalities and expressions to adopt during interactions.

Empathic interaction is initiated (Figure 1) when the observer recognizes the target’s empathic cue or behavior in a specific contextual situation. The observer, the target’s characteristics, and the relationship modulate empathic processes and outcomes. The nature of a social robot’s empathic response depends on its relationship with the human target and the situational context of interaction. Our framework clearly distinguished between the outcome (❸) and empathic response (❹) so that while a robot may “feel in” and bear an empathic outcome depending on modulating factors, it may calibrate the strength of an empathic response or even decide not to express one.

## 4. Empathy in Human–Agent and Human–Robot Interaction

Research on empathy with virtual humans or embodied conversational agents (ECAs) has been conducted with robots because it is less difficult to implement and manipulate empathy. Research on both virtual humans and robots, dubbed “advanced intelligent systems” when combined, focuses on either of the following two perspectives: (1) humans’ empathic response to the advanced intelligent system or (2) the effect of a robot’s empathic behavior on humans. The former does not necessarily involve robots that have empathic capabilities but focuses on how humans empathize with robots that have human-like characteristics. As our study is primarily interested in the latter, we only considered original studies that designed and implemented empathy in an advanced intelligent system.

Table 3 summarizes the seminar studies on empathic virtual agents. Given that all advanced intelligent systems are designed with predetermined goals, we organized the literature working backward, from the virtual agent’s goal or the study’s purpose. We then identified key elements identified in our empathy framework (Figure 1), such as *observer* and *target* characteristics, the *relationship* between the two, and the *situation* involving empathic interaction.

Empathic virtual agents have been studied in the context of playing games [73,76,78], healthcare interventions [74,77], job interviews [80], email assistance [79], social dialogue [75], or even story narration [81].

A typical empirical study investigated the effects on participants’ perceptions when interacting with or observing an empathic virtual agent compared with a non-empathic one. Overall, empathic agents were perceived positively in liking [74,75,76,81] and trust [74,76] and felt more human-like [73], caring [73,76], attractive [73], respected [74], and enjoyable [77]. Some caveats were revealed, such as participants’ negative perceptions of agents’ incongruent empathic responses [79] and participants being stressed to an empathic agent [73]. While most studies were based on one-time interaction, a few studies identified the participants’ intention to use empathic virtual agents longer [74,77]. The research community certainly has established a grounding that an empathic virtual agent, when implemented to provide an appropriate response congruent with the situation, elicits the positive perception of users for long-term interaction.

A few recent studies have taken the next step to investigate the effects of empathy modulating variables, such as the observer (virtual agent)’s *mood, personality*, and *relationship* (similarity, familiarity, liking, social bond) [75,81]. However, such manipulation was applied only to virtual agents interacting with themselves.

The *empathic tendency*, which our model defined as an individual’s predisposition for an empathic response, was implemented as an empathic threshold for a virtual agent to respond to the target’s empathic event [75]. Although the studies demonstrated that the higher the weighted means of modulating factors, the higher the level of the virtual agent’s empathy, the research did not examine the interaction effect between the factors being modulated. That is, we do not have evidence of how *liking* and *familiarity* interact. The Boukricha study [82] assigned weighted values to each variable but used the sum of such values, assuming an additive effect. A carefully designed study investigating an empathic agent with modulation compared with an empathic agent with no modulation with human participants is required. We now take a closer look in Table 4 at the empathic processes of each study.

Empathic cues were recognized from the participants’ affective states measured by facial recognition [75,77,81] or physiological measures [73,78,80]. For example, the empathy model presented by Boukricha et al. [75] assumed an emotional event when a fast and salient change occurred in the emotional state of the virtual agent. Almost all studies considered the user’s situation elicited from, for example, their multiple dialogue choices when considering the participant’s affective state.

The most sophisticated empathic processes, integrating both affective and cognitive empathic processes, were demonstrated by Rodrigues et al. [81]. The candidate emotion of the target was elicited from the facial expression (*motor mimicry*) as well as the projected emotion elicited from the appraisal of the situation (*perspective taking*). The two emotions were compared to produce a potential empathic emotion. However, the study was limited to a virtual agent emphasizing another virtual agent; therefore, follow-up studies may be conducted with human interaction.

In a strict sense, virtual agent studies only involved motor mimicry and perspective taking, but not classic conditioning, direct associations, and language associations, as outlined in Figure 1. That is, none of the studies have demonstrated conditioning scenarios (i.e., classical conditioning). No empathy research had the virtual agent store its experience and reference the agent’s past experience to elicit the emotion to them (i.e., direct associations). No study involved language-based cognitive networks engineered to demonstrate a virtual agent hearing a word and then eliciting certain emotions by triggering language-mediated associations (i.e., language associations). In short, most studies with empathic virtual agents have focused on motor mimicry and perspective taking, so a major push is required to extend our understanding of empathy in advanced intelligence systems.

Table 5 presents the seminal studies on the effects of social robots evoking empathy. Empathic social robots have been researched only in a limited domain, such as playing games or quizzes [36,83,84,85], healthcare [86], or a human’s one-way utterance to [66,87,88] or a conversation with a robot [89]. Many studies involving playing games utilized Philip’s iCat robot because of its ability to express facial expressions [36,83,84], but most recent studies utilized Softbank’s Pepper due to its multimodal capability [85,90].

Many studies on empathic social robots have game playing situations such as chess [83,84,91]. Chess is selected probably because both the participant and the robot can take turns so that the conditions can be easily tracked. Robots can also express empathic emotions based on the situation of the game. Most importantly, the participant may clearly recognize the robot expressing empathy during turns. Compared with virtual humans, research on social robots is at the stage of infancy, and participants’ perceptions of empathic robots are limited to perceived trust [83], human-likeness [87], friendliness [90,91], social presence, and engagement [84,85]. However, the most recent HRI research applied an advanced computational model (e.g., deep learning) to model empathy [89,90], which merits more discussion in Section 5. We now take a closer look at the empathic processes of each study in Table 6.

Interestingly, while most studies on both virtual agents and empathic robots, involving motor mimicry, had an intelligent system to respond with the same modal (i.e., facial mimicking), Hegel’s study [92] involves cross-modal motor mimicry. That is, when participants read an emotional story to a robot, it responded with a congruent emotional expression on its face. The idea of cross-modal mimicry is grounded by Chovil [93]. More sophisticated research will face the question of how to combine empathic cues from different modalities and combine them into a singular representative value. We defined this cross-modality empathic feature as one of the key features of the Type III Social Robot.

Overall, research on empathic virtual agents and social robots seems to elicit a human’s positive response when a robot’s empathic response is expressed and the outcome is congruent with the situation. Perceived measurements that may gain people’s interest in long-term interactions, including liking, trust, attractiveness, engagement, enjoyment, believability, and human-likeness, had a positive effect with an empathic virtual agent or a robot. Surprisingly, except for Cramer et al. [83], none of the studies measured perceived empathy directly. We suggest referencing established perceived empathy measures, such as the relationship inventory measures by Barrett-Lennard [94]. The relationship inventory measures possessed adequate internal reliability, and the scales fit the kind of interaction with a social robot. A few scales can be slightly modified to the HRI, such as “the robot tries to understand me from my point of view”, a scale measuring cognitive empathy, or “the robot does not understand the way I feel”.

## 5. Synthesis and Discussion

Developing an empathic robot, like all other robots, requires a concrete function definition that serves the purpose of the robot. Based on such functions, designers may design interaction scenarios, and engineers may develop the software and hardware architecture of the robot.

One consideration is the purpose of the robot. Is the robot to assist passengers at airports? Is it a companion robot built for long-term interactions? Is it a counseling robot that provides health-related advice? This purpose defines the level and type of empathic capabilities. The demanded capability requires a number of empathic characteristics.

**Figure 2:**
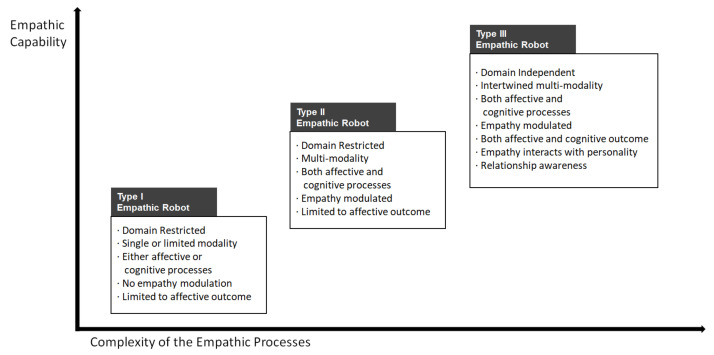
Empathic capability as a function of the complexity of empathic processes.

We grouped the three types of empathic robots that organized such characteristics (see Figure 2). Generally, the more applicability in terms of domains and tasks, more sophisticated empathic robots (i.e., Type III robots) are required. Currently, most empathic research on social robots is slowly moving to Type II robots, and we are starting to see Type I empathic robots in the industry. As HRI research precedes commercialization, we expect to see research on Type III robots in the near future.

We do not suggest a full-fledged empathic robot (Type III) for all situations. Certain tasks may not require sophisticated empathic capabilities. Depending on the tasks, users may perceive empathic responses negatively [95]. The exact areas in which users require empathy is an important research question to be illuminated.

As depicted in Figure 2, the following factors need to be considered when designing an empathic robot. As research on empathic social robots is at the stage of infancy, certain factors may lack empirical evidence.

### 5.1. Domain-Dependent vs. Domain-Independent

All social robots in the literature are heavily domain-dependent. For example, a social robot may be exclusively designed for educational purposes. A robot cannot function outside the designed application. That is, currently there is no empathic robot that can switch tasks and service multiple domains. For example, we are yet to see an empathic robot that serves coffee at work and then interacts with family members after work. As reviewed (see Figure 1), the empathic event may influence the likelihood of empathizing [69] and the power of empathic responses [20]. A domain-independent robot should seamlessly manipulate its *empathic tendency* (i.e., a robot’s predisposition to engage in an empathic interaction) according to the situation at hand.

One reason why domain-independent empathic robots (i.e., Type III empathic robots) are difficult to produce is that, with the current AI technology involving voice recognition and natural processing, all use cases should be defined and trained for a robot to recognize. Designers should carefully describe all possible interaction scenarios as well as the kind of empathy process to engineer. For example, should we engineer empathy for a robot that serves coffee? If so, to what extent? Some customers may not necessarily expect empathy.

### 5.2. Emotion Modeling

Our definition of empathy for social robots involves the robot’s (*observer*) capability and process to recognize the human’s (*target*) emotional state, thoughts, and situation and to produce affective or cognitive responses. Although there are exclusive cognitive responses involving interpersonal accuracy and attributional judgment, almost all resulted in affective responses, either parallel or congruent. Only Prendinger’s study [96] involved an exclusive cognitive response.

As empathy involves emotions, designers should define the kind of emotion that may occur in a given scenario for both the observer and the target. If emotion recognition and expression require sophistication, an emotion model is required. Existing research is not clear regarding which model is more effective or may gain a more positive perception of the social robot by the participant.

### 5.3. Single vs. Multimodality

Most studies have focused on single modal interaction with empathic robots, but we are now seeing an increasing number of multimodal robots. Multimodal recognition is powerful because the robot recognizes redundant cues for a better understanding of the user’s affective state and thoughts. Owing to situational reasons, users tend to choose one modality over another. If the robot does not support such a channel, the opportunity to recognize its empathic cue is lost. The most advanced Type III robot may combine multimodal channels to maximize empathic interactions. This is what humans do as well.

### 5.4. Empathy Modulation

As we have reviewed in Figure 1, given the same empathic cues, the empathy outcomes differ by modulating variables including the situation (strength and characteristics) and relationship (similarity, familiarity, liking, and social bond). Designers should possess a good understanding of tasks and contexts. Are there multiple users involved? Should the empathic robot respond differently to different users with various relationships? When empathic cues differ in strength, how should empathy change?

### 5.5. Affective and Cognitive Outcomes

Although nearly all social robot research is limited to either an affective outcome (parallel response to the target) or a cognitive outcome, many virtual agents have virtual agents for producing both outcomes. We want to emphasize that the outcome is not necessarily expressed to users. For example, an empathic robot may feel a certain way that is represented by its emotion model but decide not to exercise restraint in its feelings because of certain reasons; humans do this all the time. The Type III empathy robot should engineer this control module that moderates its emotions and empathic expressions.

### 5.6. Interaction with Personality

Currently, there is no research on the effect of differential personality on the empathic capability of a social robot. However, emotions are intertwined with personality. As we have seen in the case of AI assistants, more customers require customization of their assistants (e.g., voice personas), so there is a need for different personalities to modulate the empathic process.

### 5.7. Anthropomorphic versus Biomorphic

According to Bartneck and Forlizzi [97], social robots can be classified as anthropomorphic (i.e., mimicking a human) or biomorphic (i.e., mimicking a lifelike object). Interestingly, the two most used robotic platforms for an empathic study are biomorphic (i.e., cat-like) iCat [36,83,84] and anthropomorphic Pepper [85,90]. We have not seen any studies on how different forms affect empathy. Future studies may address this.

### 5.8. For a Hybrid Computational Model

Yalcin and DiPaola [98] recently reviewed computational models in artificial agents. They argued that the data-driven approach to model empathy is in its infancy. This is consistent with our findings that few HRI studies on deep learning-based computational models were published only recently in 2020 [90] and 2021 [89], respectively. Meanwhile, conceptual models (i.e., Figure 1) based on empathy theories would provide a useful framework because they outline mechanisms involving empathy and include solid explanatory power [98]. Eventually, we expect a hybrid computational model where data are gathered, analyzed, and predicted (i.e., data-driven) based on the sub-components envisioned by the theory-driven model.

In this article, we reviewed all prominent empathy theories and established a conceptual framework that illuminates critical components to consider when designing an empathic robot, including the empathy process, outcome, and the *observer* and *target* characteristics. This model is complemented by empirical research involving empathic virtual agents and social robots. We identified many gaps in the current literature on empathic social robots and overviewed essential factors to be included in a computational model. We also suggest that critical factors such as domain dependency, multi-modality, and empathy modulation be considered when designing, engineering, and researching empathic robots.

## Figures and Tables

**Table 1:** The number of articles screened, assessed, and included for a review.

	Interpersonal	Human–Agent	Human–Robot
Abstract Screened	1116	128	76
Full-text Assessed	232	27	21
Studies Included	70	10	12

**Table 2:** Definitions of empathy.

Emphasis on	Author(s)	Definition
Affective	[26]	“The vicarious experiencing of an emotion that is congruent with, but not necessarily identical to, the emotion of another individual (p. 146).”
		“One specific set of congruent emotions, those feelings that are more other-focused than self-focused.”
	[27]	“An affective response that stems from the apprehension or comprehension of another’s emotional state or condition, and which is similar to what the other person is feeling or would be expected to feel (p. 71).”
	[28]	“Consists of a sort of ‘mimicking’ of one person’s affective state by that of another.”
	[2]	“An affective response more appropriate to another’s situation than one’s own (p. 4).”
	[29]	“Feeling what another person feels because something happens to them which does not require understanding another’s internal states (p. 411–412).”
Cognitive	[30]	“The imaginative transposing of oneself into the thinking, feeling, and acting of another (p. 343).”
	[31]	“A form of complex psychological inference in which observation, memory, knowledge, and reasoning are combined to yield insights into the thoughts and feelings of others (p. 2).”
	[32]	“Ability to put yourself in the other person’s position, establish rapport, and anticipate his reaction, feelings, and behaviors (p. 269).”
Affective and Cognitive	[33]	“The capacity to understand and enter into another person’s feelings and emotions or to experience something from the other person’s point of view (p. 248).”
	[34]	“A set of constructs having to do with the responses of one individual to the experiences of another. These constructs include the processes taking place within the observer and the affective and non-affective outcome which result from those processes (p. 12).”
	[35]	“The capacities to resonate with another person’s emotion, understand his/her thoughts and feelings, separate our own thoughts and emotions from those of the observed and responding with the appropriate prosocial and helpful behavior (p. 201).”

**Table 3:** Studies on empathic virtual agents.

Author	Purpose	Observer	Target	Relationship	Situation	Results
[73]	To increase the level of social engagement	Agent competitor with neutral, self-centered, empathy condition	Participant competitor motivated for monetary reward when won	Competitive power relationship—fear and anger	Cards game *Skip-Bo*	Participants in empathic conditions felt less lonely, perceived the agent as more caring, attractive, and more human-like but more stressed
[74]	To improve long-term relationship quality	Exercise advisor with or without empathic relationship building skills	Exercise client	Advisor–client	Daily conversation on target’s physical activity for a month	Participants respected, liked, and trusted the empathic agent more and wished continued interaction
[75]	To understand factors modulating agent’s empathic behavior	Agent EMMA with *mood* varied	Agent MAX	*Liking* and *familiarity* were varied between virtual agents	Three-way conversation among EMMA, MAX, a participant	Participants liked the agent that empathizes the other agent more
[76]	For the positive perception of agents	Agent (photographic human face) game player with self-oriented or empathy condition	Participant game player	Co-present gamer (not a competition)	Each plays blackjack with a dealer (split-screen)	Participants liked, trusted, and perceived caring, and felt more supported by the empathic agent
[77]	To change health-related behaviors (alcohol consumption, exercising, drug use)	3D personalized on-demand virtual counselor	Participant counselee	Counselor–counselee	Behavioral change in health interventions on excessive alcohol consumption	Participants accepted and enjoyed the empathic agent more and showed an intention to use the system longer
[78]	To investigate the effects of parallel and reactive virtual agent responses	Six agents with a *reactive* or *parallel* response	Member of a research team on an island	Inhabitant—researcher	Participants solve a mystery on an island while interacting with agents	A model was induced from positively perceived agent responses in terms of appropriateness and effectiveness
[79]	To investigate the effects of dialogue agent with beliefs, uncertainties, and intentions	Expressive 3D taking head with empathic or non-congruent empathic condition	Email user	Assistant—user	Participants converse with an agent to find out information on their mail (sender, message)	Participants perceived the non-congruent agent more negatively
[80]	To support job-seekers preparing for an interview by reducing their stress levels	Mail companion agent in a suit invisible to the interviewer agent	Participant Interviewee	Companion	Job Interview	Participant’s stress level was reduced by empathic feedback
[81]	To establish a generic computational model of empathy	Four virtual agents interacting (can be either an observer or a target) varied in *mood, personality*	Four virtual agents	Relationships among agents were varied in *similarity, social bond, liking*	A short narrative consists of virtual agents interacting (compliment, criticize) at a schoolyard	Participants evaluated virtual agent-agent interactions from a video. They perceived virtual agents applied with an empathy model more positively, especially with an agent who carried out a prosocial behavior (comforting)

**Table 4:** Empathic recognition and responses of virtual agent studies.

Author	Empathy Recognition	Process	Outcome	Empathy Responses
[73]	Affective states - Physiological data (skin conductance, EMG) Situation - User actions (moves in game *Skip-Bo)*	Cognitive - Assumes whether the participant is happy or distressed	Affective - Parallel (positive), reactive (sympathy) Cognitive - Estimation of feelings	Facial expression and nonverbal voice (grunts, moans)
[74]	Situation - Dialogue (multiple choices)	Cognitive	Affective Cognitive	TTS voice (“I’m sorry to hear that”), synchronized hand gestures, posture, gaze
[75]	Affective states - Facial expression of the virtual human MAX Situation - Dialogue (praise, insult)	Affective - Motor mimicry (shared representation system)	Affective - Parallel, reactive	Facial expression, speech prosody, verbal utterance
[76]	Situation - User actions (the out come of each round of blackjack)	Cognitive	Affective - Parallel Cognitive - Estimation of feelings	Facial expression
[77]	Affective states - Facial expression Situation - Dialogue	Affective - Motor mimicry (head posture) Cognitive - Perspective taking	Affective - Parallel, reactive Cognitive	TTS voice, nonverbal (head nod, direction)
[78]	Affective states - Selecting emotions when asked Galvanic skin response, heart rate Situation - The context in the island narrative	Affective Cognitive	Affective - Parallel, reactive	One or two sentences of text responses
[79]	Situation - Dialogue (multiple choices)	Cognitive	Affective - Congruent, incongruent	Facial expression, text responses
[80]	Affective states - Physiological data (skin conductance, EMG) Situation - Dialogue (multiple choices)	Cognitive	Cognitive - Interpersonal accuracy	Text responses (“It seems you did not like this question so much.”)
[81]	Affective states - Facial expression Situation - Self-projection appraisal	Affective Cognitive	Affective Cognitive	Facial expression, text responses

**Table 5:** Studies on empathic robots.

Author	Purpose	Observer	Target	Relationship	Situation	Measures and Results
[83]	To investigate attitudes toward a robot with accurate or inaccurate empathy	A robot with a synthetic female voice	A male user	Collaborator	A male user and a robot played an online collaborative game.	Participants viewed a video of a robot emphasizing a user. Their trust decreased when the robot’s empathic responses were incongruent with the user.
[87]	To evaluate the acceptance of mimicked emotion	A robot mimics the target’s voice and does facial expressions with parallel emotion	Human participant	Not defined	Participants read an emotion-embedded story.	Participants perceived the robot’s mimicking response to be more adequate and human-like than the neutral response.
[36]	To investigate the effects of robot’s empathic responses when the relationship was varied	A robot reacts to the player’s chess moves empathically to a player and neutrally to the other	Two participants	Relationship between the robot and each participant was varied	Two humans played chess.	Participants perceived the empathic robot as being friendlier than the non-empathic one.
[84]	To evaluate an empathic model for social robots interacting with children	A robot reacts to the children’s chess move based on the empathic appraisal of children’s affect and the game’s state	Children	Not defined	A child played chess against the robot.	Participants responded positively in social presence, engagement, help, and self-validation when interacting with a robot and remained similar after five weeks.
[66]	To understand human’s perception of the robot’s imitation	A robot with a full head gesture mimicking, partial mimicking (nodding), and non-mimicking	Human participant	Not defined	Participants described non-emotional personal statements and salient personal experience.	Male participants made more gestures than women while interacting with the robot. Participants showed coordinated gestures (co-nodding).
[86]	To understand human’s perception of robot speech	A robot conversed with the participants in three situations (greeting, medicine reminder, guiding the user to use the touch interface)	Human participant	Not defined	Participants interacted with a Healthbot as a patient.	Participants were able to perceive empathy and emotions in robot speech. They preferred it over the standard robotic voice.
[89]	To develop a deep learning model for a social robot that mirrors humans	A robot with a display that animates facial expressions	Human participant	Not defined	Participants conversed with the robot with various facial expressions.	Participants’ interaction data were used to train the model.
[90]	To evaluate a robot with an empathy model that simulates advanced empathy (i.e., reactive emotions)	A robot (Pepper) embedded with the proposed Autonomous Cognitive Empathic Model that expresses parallel and reactive emotions	Human participant	Not defined	Participants watched emotion-eliciting videos on the robot’s tablet and interacted with the robot.	Participants’ responses were better to a robot embedded with the proposed model than the baseline model in terms of social and friendship constructs.
[88]	To evaluate a robot with a deep hybrid neural model for multimodal affect recognition	A robot embedded with a model that simulates intrinsic emotions (i.e., mood)	Human Participant	Not defined	Participants told a story portraying different emotional contexts to the robot.	Independent annotators rated the robot higher in performance (i.e., the accuracy of empathic emotion) than the participants.
[85]	To evaluate a robot with an empathy model that draws participant’s attention when inattentive	A robot (Pepper) embedded with the attention-based empathic module to hold participant’s attention	Human participant	Not defined	Participants responded to a quiz on the robot’s tablet.	Participants perceived the empathic robot as more engaging and empathic and as spending more time than the non-empathic robot.

**Table 6:** Empathic recognition and responses in social robot studies.

Author	Empathy Recognition	Process	Outcome	Empathy Responses
[83]	Situation - Win or lose the game	Cognitive	Affective Cognitive	Facial expression, verbal responses
[87]	Affective states - Speech signal	Affective - Motor mimicry	Affective - Parallel	Facial expression
[36]	Situation - Appraisal of each move	Cognitive - Perspective taking	Affective Cognitive	Facial expression, verbal responses
[84]	Affective states - Facial cues, head direction Situation - Appraisal of each move	Cognitive - Perspective taking	Affective Cognitive	Facial expression, verbal responses
[66]	Affective states - Facial expression	Affective - Motor mimicry	Affective - Parallel	Facial expression
[86]	Situation - e.g., time to take medicine	Cognitive	Affective Cognitive	Verbal responses (The robot’s facial expression was not varied with emotions.)
[89]	Affective states - Facial expression	Affective - Motor mimicry	Affective - Parallel	Facial expression
[90]	Affective states - Facial expression	Affective Cognitive - Perspective taking	Affective - Parallel Cognitive - Reactive	Facial expression, verbal responses, gestures
[88]	Affective states - Facial expression - Speech signal	Affective	Affective - Parallel	Facial expression
[85]	Situation - Participant’s attentiveness	Cognitive	Affective	Facial expression, verbal responses, gestures
